# Prostate transglutaminase (TGase-4, TGaseP) enhances the adhesion of prostate cancer cells to extracellular matrix, the potential role of TGase-core domain

**DOI:** 10.1186/1479-5876-11-269

**Published:** 2013-10-25

**Authors:** Wen G Jiang, Lin Ye, Andrew J Sanders, Fiona Ruge, Howard G Kynaston, Richard J Ablin, Malcolm D Mason

**Affiliations:** 1 Metastasis and Angiogenesis Research Group, Cardiff University School of Medicine, Heath Park, Cardiff CF14 4XN, UK; 2 Department of Pathology, Health Sciences Center, University of Arizona College of Medicine and the Arizona Cancer Center, 1501 N. Campbell Avenue, P.O. Box 245043, Tucson, AZ AZ 85724-5043, USA

**Keywords:** Transglutaminase, Transglutaminase-4, Cell-matrix adhesion, Focal adhesion kinase, Paxillin, Integrins, Electric cell sensing, Prostate cancer

## Abstract

**Background:**

Transglutaminase-4 (TGase-4), also known as the Prostate Transglutaminase, is an enzyme found to be expressed predominately in the prostate gland. The protein has been recently reported to influence the migration and invasiveness of prostate cancer cells. The present study aimed to investigate the influence of TGase-4 on cell-matrix adhesion and search for the candidate active domain[s] within the protein.

**Methods:**

Human prostate cancer cell lines and prostate tissues were used. Plasmids that encoded different domains and full length of TGase-4 were constructed and used to generate sublines that expressed different domains. The impact of TGase-4 *on in vitro* cell-matrix adhesion, cell migration, growth and *in vivo* growth were investigated. Interactions between TGase-4 and focal adhesion complex proteins were investigated using immunoprecipitation, immunofluorescence and phosphospecific antibodies.

**Results:**

TGase-4 markedly increased cell-matrix adhesion and cellular migration, and resulted in a rapid growth of prostate tumours *in vivo*. This effect resided in the Core-domain of the TGase-4 protein. TGase-4 was found to co-precipitate and co-localise with focal adhesion kinase (FAK) and paxillin, in cells, human prostate tissues and tumour xenografts. FAK small inhibitor was able to block the action mediated by TGase-4 and TGase-4 core domain.

**Conclusion:**

TGase-4 is an important regulator of cell-matrix adhesion of prostate cancer cells. This effect is predominately mediated by its core domain and requires the participation of focal adhesion complex proteins.

## Background

Prostate transglutaminase, also known as transglutaminse-4 (TGase-4), is a member of the transglutaminase [EC 2.3.2.13] family. Similar to some of the members, such as keratinocyte TGase, TGase-4 has a relatively restricted pattern of distribution in the body, namely, confined to the prostate gland [1-3]. The role of TGase-4 is not entire clear. Although early studies, mostly using a single technique, have shown that TGase-4 may be reduced in prostate cancer, in comparison with normal prostate tissues [4,5], recent studies have indicated otherwise. Most interestingly, we and others have recently shown that levels of TGase-4 in prostate cancer cells may be linked to the aggressiveness of the cells. For example, over-expression of TGase-4 in prostate cancer cells increases the invasiveness and the migration of prostate cancer cells. Vice versa, knocking down TGase-4 from TGase-4 positive prostate cancer cells rendered the cells less aggressive [6]. Furthermore, levels of TGase-4 in prostate cancer cells are amongst factors that influence the cell’s response to other molecules, namely MDA-7/IL-24 and HGF-L/MSP-1 [7,8].

The influence of TGase-4 on cell invasiveness and migration is not an isolated observation in the TGase family. For example, tissue TGase (TGase-2) has been shown to be a possible coreceptor for cancer cell-matrix adhesion and that impairment of TGase-2 increases the adhesion to matrix and migration over matrix [9]. TGases have been implicated in the development of cancer and metastasis [10]. TGase-2 was found to exist at much higher levels of drug resistant cancer cells and in patients who developed drug resistance [11]. TGases are also involved in regulating apoptosis [12], which may be linked to the fact that TGase-2 is a caspase substrate during apoptosis [13] and a substrate of Calpain [14]. Another calcium regulator, psoriasin (S100A7) is also a substrate of TGase-2 [15].

Matrix invasiveness and the migratory ability of cancer cells are associated with a number of extracellular and intracellular events. Critical to the invasion and migration of cancer cells is cell-matrix adhesion [16,17]. Cell-matrix adhesion is an essential cellular function in pathophysiological processes of the body; and as previously established in the past decades, cell-matrix adhesion is largely mediated by a group of transmembrane proteins, namely integrins which are formed by the heterodimeric combination of subunit proteins (alpha- and beta- units). The interaction between integrins and the extracellular matrix not only provides a mechanical mechanism for cells to be attached to matrix and the body structure, it is also essential to mediating the signaling of the cells, allowing communication between the extracellular and intracellular environment.

The intriguing role of TGase-4 in prostate cancer cells, namely the involvement in the invasion and motility, indicated that a potential link underlying this critical function of the enzyme may be cell-matrix adhesion. Here, we report that TGase-4 is indeed involved in the matrix adhesion of prostate cancer cells. This action of TGase-4 appears to rely on the TGase-4 core domain and potentially via the FAK pathway. Knowledge of this molecular and cellular link may prove useful in consideration of the development of therapeutic modalities for prostate cancer.

## Methods

### Materials and cell lines

Human prostate cancer cells, PC-3 and CA-HPV-10 were from ATCC (American Type Cell Collection, Manassas, VA, USA). Fresh frozen human prostate tissues (normal and tumour), were collected from University Hospital of Wales under the approval of local ethical committee (South East Wales Local Research Ethics Committee, Protocol Number - 03/5048). Written consents were obtained from the patients. Tissues were obtained immediately after surgery and stored at -80°C until use.

Monoclonal antibodies of human FAK and paxillin were from Transduction laboratories and a neutralising monoclonal antibody to β1-integrin was obtained from R&D Systems Europe (Abbingdon, England). Phospho-specific antibody to FAK and paxillin were from Santa Cruz Biotechnologies, Inc. Rabbit and rat anti-human TGase-4 antibodies were from Abcam (Cambridge, England, UK), Santa-Cruz Biotechnologies Inc., (Santa Cruz, CA, USA) and Abnova (Taipei, Taiwan), respectively. Recombinant human TGase-4 [rhTGase-4] was from Abnova (Taipei, Taiwan). Fluorescence and HRT-conjugated secondary antibodies were from Sigma-Aldrich [Poole, Dorset, England, UK]. Small inhibitor to FAK [FP-573228] was from Tocris Biochemicals (Bristol, England, UK) and Santa Cruz Biotechnologies, Inc., (Santa-Cruz, California, USA], respectively. Monoclonal antibody to phosphotyrosine (PY20), monoclonal anti-GAPDH and protein A/G agarose were from Santa-Cruz Biotechnologies, Inc. (Santa Cruz, CA, USA). Recombinant human hepatocyte growth factor/scatter factor (HGF/SF) was a gift from Dr. T. Nakamura (Osaka University Medical School, Osaka, Japan). Matrigel (reconstituted basement membrane) was purchased from Collaborative Research Products (Bedford, Massachusetts, USA). Transwell plates equipped with a porous insert [pore size 8 μm] were from Becton Dickinson Labware (Oxford, UK). DNA gel extraction and plasmid extraction kits were from Sigma-Aldrich (Poole, Dorset, England, UK). All other chemicals were from Sigma-Aldrich (Poole, Dorset, England, UK) unless stated otherwise.

### Construction of hammerhead ribozyme transgenes targeting the human prostate-transglutaminase and mammalian expression vector for human prostate transglutaminase (TGase-4)

Hammerhead ribozymes that specifically target a GTC site of the human prostate TGase-4 (GenBank accession NM_003241), based on the secondary structure of TGase-4, have been generated as previously described [6]. Touch-down PCR was used to generate the ribozymes with the respective primers [Additional file 1]. This was subsequently cloned into a pEF6/V5-His vector (Invitrogen, Paisley, Scotland, UK; selection markers: ampicillin and blasticidin, for prokaryotic and mammalian cells, respectively). After identification of the colonies with correct inserts using direction specific PCR, the colony was amplified. Following purification and verification, the extracted plasmids were subsequently used for transfecting prostate cancer cells by way of electroporation (Easyjet, Flowgen, England, UK). Following selection of transfected cells with blasticidin (used at 5 μg/ml) and verification, the following stably transfected cells were established: TGase-4 knock-down cells [designated here as CA-HPV-10^ΔTGase4^ in this manuscript], plasmid only control cells (CA-HPV-10^pEFa^), and the wild type, CA-HPV-10^WT^. The CA-HPV-10^ΔTGase4^ and the CA-HPV-10^pEFa^ cells thus created were always kept in a maintenance medium which contained 0.5 μg/ml blasticidin.

Full length human TGase-4 coding region was amplified from a cDNA library of human prostate tissues using primers listed in Additional file 1. Reverse transcription was carried out using a RT kit (Sigma-Aldrich, Poole, Dorset, England, UK) and amplification using an extensor PCR master mix which has an additional proof reading polymerase (AbGene, Ltd., Surrey, England, UK). The TGase-4 full length coding product was similarly cloned into the pEF6 vectors. PC-3 cells which express little TGase-4 were transfected with either the control vector or TGase-4 expression vector. Stably transfected cells were designated as PC-3^pEF/His^ and PC-3^TGase4exp^, for control transfection and TGase-4 expression, respectively. In subsequent experiments, the mixture of multiple clones for each stably transfected cells were used.

### Creation of sublines of PC-3 cells which expressed mutant TGase-4

The following TGase-4 mutant constructs were generated from human prostate cDNA library: TGase-N domain deleted (TG4ΔN, amino acids 8–123 deleted), TGase-C domain deleted [TG4ΔC, amino acid 585–684 deleted], TGase-core expression only (TG4CoreLarge, containing amino acids 124–585), and TGase-core central region (TG4CoreSmall, containing amino acids 260–353), TGase-4 N-domain only (TG4ΔN/core) and TGase-4 C-domain only (TG4ΔC/core), using the pEF6 vector. Primers used are listed in Additional file 1. PC-3, negative for TGase-4 was transfected with the plasmids with mutant TGase-4 and selected and verified for the expression of mutant TGase-4 by way of RT-PCR. Stably transfected cells, designated: PC3^TG4ΔN^, PC3^TG4ΔC^, PC3^TG4ΔN/core^, PC3^TG4ΔC/core^, PC3^TG4CoreLarge^ and PC3^TG4CoreSmall^ were used in the subsequent assays.

### RNA preparation and RT-PCR

RNA from cells was extracted using an RNA extraction kit (AbGene Ltd., Surrey, England, UK) and concentration quantified using a spectrophotometer (Wolf Laboratories, York, England, UK). cDNA was synthesised using a first strand synthesis with an oligo^dt^ primer (AbGene, Surrey, UK). The polymerase chain reaction (PCR) was performed using sets of primers (Additional file 1) with the following conditions: 5 min at 95°C, and then 20 sec at 94°C-25 seconds at 56°C, 50 sec at 72°C for 36 cycles, and finally 72°C for 7 min. ß-actin was amplified and used as a house keeping control. PCR products were then separated on a 0.8% agarose gel, visualised under UV light, photographed using a Unisave^tm^ camera [Wolf Laboratories, York, England, UK] and documented with Photoshop software.

### Quantitative analysis of tranglutaminase

The level of the prostate TGase transcripts in the above-prepared cDNA was determined using a real-time quantitative PCR, based on the Amplifluor^TM^ technology that was modified from previous reported [18]. Briefly, pairs of PCR primers were designed using the Beacon Designer^tm^ software (version 2, Palo Alto, California, USA) (Additional file 1), but added to one of the primers was an additional sequence, known as the Z sequence (5′actgaacctgaccgtaca′3) which is complementary to the universal Z probe (Intergen Inc., Oxford, England, UK). The reaction was carried out using the following: Hot-start Q-master mix (AbGene, Ltd., Poole, Dorset, England, UK), 10 pmol of specific forward primer, 1 pmol reverse primer which has the Z sequence (underlined) (Additional file 1), 10 pmol of FAM-tagged probe, and cDNA generated from approximately 50 ng RNA. The reaction was carried out using IcyclerIQ^tm^ (Bio-Rad, Hammel Hemstead, England, UK) which was equipped with an optic unit that allows real time detection of 96 reactions. The following condition was used: 94°C for 12 min, 50 cycles of 94°C for 15 sec, 55°C for 40 sec and 72°C for 20 sec. The levels of the transcripts were generated from an internal standard that was simultaneously amplified with the samples.

### In vitro cell growth assay

This was based on a previously reported method [19]. Cells were plated into 96-well plated at 2,000 cells/well followed by a period of incubation. Cells were fixed in 4% formaldehyde at the day of plating and daily for the subsequent 5 days. 0.5% crystal violet (w/v) was used to stain cells. Following washing, the stained crystal violet was dissolved with 10% (v/v) acetic acid and the absorbance was determined at a wavelength of 540 nm using an ELx800 spectrophotometer. Absorbance represents the cell number.

### Cell-matrix adhesion assay

This was based on a previously reported method [19]. Briefly, tissue culture 96-well plates (Greiner Laboratories, Gloucester, England) were precoated with 5 μg Matrigel. After rehydration of the well with Matrigel, 10,000 cells [transfected and controls] were added to each well. After incubating the plates for 40 min in an incubator, culture medium and non-adherent cells were disregarded. The plates were then washed 5 times with a sterile BSS buffer and added with 4% formalin for more than 30 min. 0.5% of crystal violet was used to stain the cells. After washing, the number of cells adhered to Matrigel coated surface was counted under a microscope and is shown here as the number of adherent cells per field.

### Electric cell-substrate impedance sensing (ECIS) based cell adhesion assay

ECIS Zθ model was used in the present study and for cell modelling. Cells were monitored at 1,000, 2,000, 4,000, 8,000, 16,000, 32,000 and 64,000Hz. The adhesion was analysed by the integrated Rb modelling method [20-22].

### Immunoprecipitation and western blotting

Cells were grown in 25 cm^2^ flasks and removed by cell scrapper. After centriguation (2000 rpm), media were removed and cell pellets were lysed using a lysis buffer (50 mM Tris, 150 mM NaCl, pH 8.0, with the following addition: 1% Triton, 0.1% SDS, 2 mM CaCl_2_, 100 μg/ml phenylmethylsulfonyl fluoride, 1 μg/ml leupeptin, 10 mM sodium orthovanadate and 1 μg/ml aprotinin). Fresh frozen human prostate tissues, Normal and Tumour, were homogenised in a HCMF buffer. Proteins from cells and tissues were quantified, diluted to same concentration, and mixed with sample buffer before boiling. For phosphorylation study, cells were subject to serum hunger for 2 hrs, before rhTGase-4 was added. Medium alone, medium with control buffer, BSS plus 0.1% BSA, or Sodium orthovanadate (1 mM with 0.1% H_2_O_2_) were used as the respective negative and positive control. After one hour, cells were harvested and lysed. To each cell lysate was added anti-FAK, anti-paxillin, anti-integrin-ß1, or anti-TGase-4 antibodies. After the immunocomplex was precipitated using protein A/G agarose, the protein was separated on 8% SDS PAGE and the respective phosphorylated bands probed with anti-phosphotyrosine antibody (PY20) and potentially co-precipitated TGase-4 was probed with anti-TGase-4 antibody. For the protein interaction analysis, protein lysates from TGase-4 positive CA-HPV-10 cells and from prostate tissues were similarly added. The antibodies for immunoprecipitation and the precipitate were similarly probed by anti-TGase-4 antibody. GAPDH was used as loading control.

### In vivo tumour model

*In vivo* studies were reviewed by Biological Standard and Experimental Animal Application Ethics Committee of Cardiff University and conducted under the British Home Office project license (PIL 30/5509 and PPL 30/2591). Animal Welfare were fully observed in accordance with the United Kingdom Coordinating Committee for Cancer Research (UKCCCR) guidelines for the welfare of animals in experimental neoplasia (http://www.ncrndev.org.uk).

Athymic nude mice (CD-1, Charles River Laboratories) were injected via subcutaneous route, prostate cancer cells (control and TGase-4 transfected) at 0.5 million per 100 μl solution which contained 2 mg/ml Matrigel (n = 6 per group). Tumours were monitored weekly for a period of 4 weeks. The size of tumours were measured using a digital caliper. The volume of tumours were calculated by lengthxwidthx0.54. At the end of the experiments, tumours were dissected and stored at -80°C and subsequently processed for molecular and histological analysis.

### Immunofluorescence staining of TGase-4, FAK, paxilliln and β1-integrin in cells and tissues

Frozen sections of prostate tissues (normal and tumour) and tumour xenografts were cut at a thickness of 6 μm using a cryostat. The sections were mounted on super frost plus microscope slides, air dried and then fixed in a mixture of 50% Acetone and 50% methanol. The sections were then placed in “Optimax” wash buffer for 5 –10 min to rehydrate. Sections were incubated for 20 min in a 1% horse serum blocking solution and probed with the primary antibodies (anti-FAK, anti-Paxillin and anti-integrin at 1:400, anti-TGase-4 at 1:250 dilutions). Following extensive washings, sections were incubated for 30 mins in the secondary FITC- and TRITC conjugated antibodies (1:1,000) in the presence of Hoescht33258 at 10 μg/ml (Sigma-Aldrich, Poole, Dorset, England, UK). For dual immunofluorescence staining, mouse monoclonal anti-FAK, Paxillin or integrin was added together with rabbit anti-TGase-4 antibody. Secondary antibodies were TRITC-conjugated anti-mouse IgG and FITC-conjugated anti-rabbit IgG mixture. Following extensive washings, the slides were mounted using Flurosave^tm^ mounting media (Calbiochem, Nottingham, UK) and allowed overnight in fridge to harden, before being examined. Slides were examined using a Olympus fluorescence microscope and photographed using a Hamamatsu digital camera. The images were documented using the Cellysis software (Olympus). Photoshop CS6 was used to produce a merge image from the dual stained images.

Statistical analysis was carried out using SigmaPlot (version 11). Mann–Whitney U test or ANOVA on rank, and Student’s “t” test were respectively used for skewed and abnormally distributed data.

## Results

### Manipulation of TGase-4 in prostate cancer cells

**Figure 1:**
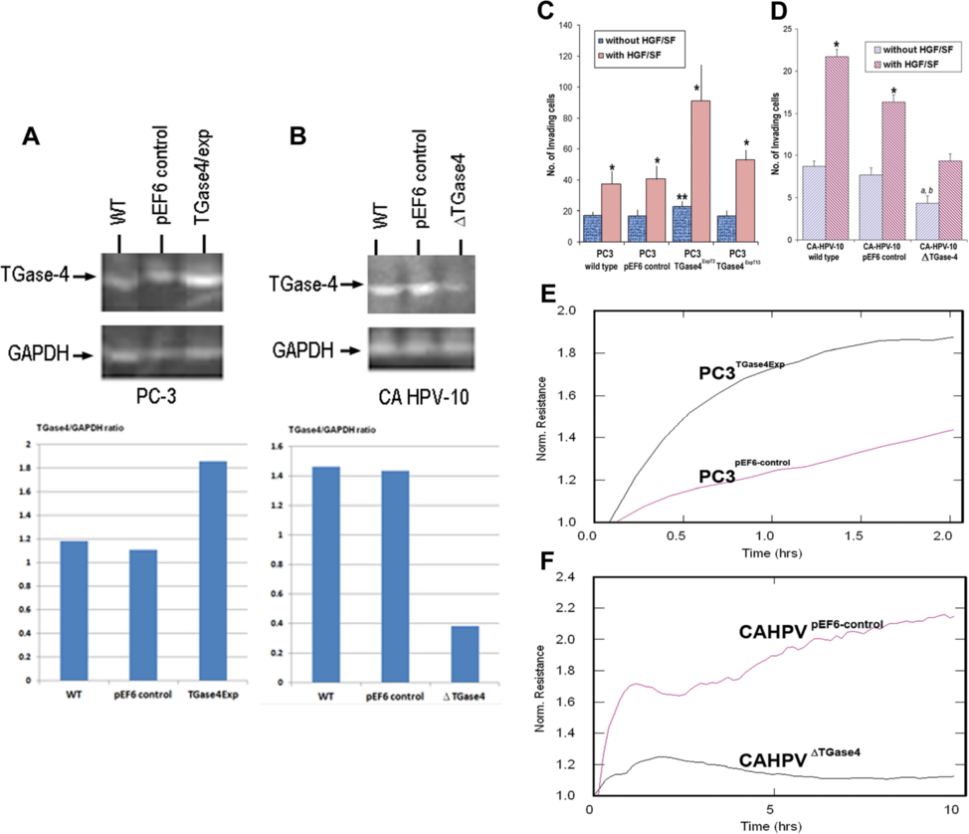
**Effects of TGase-4 expression and cell-matrix adhesion of prostate cancer cells. A** and **B**: Western blotting analysis of protein expression of TGase-4 after transfections for PC-3 **(A)** and CA HPV-10 **(B)** cells. Bottom panel is the TGase-4/GAPDH ratio. **C**: Over-expression of TGase-4 in PC-3 cells signficantly increased matrix adhesion. *p < 0.05 vs no HGF, ** p < 0.05 vs control cells. **D**: Effects of TGase-4 knockdown on the *in vivo* invasiveness of CA-HPV-10 cells. Reduction of TGase-4 significantly reduced the invasiveness of the prostate cancer cells. *p < 0.05 vs no HGF, ** p < 0.05 vs control cells. **E** and **F**: ECIS based analysis of matrix adhesion of PC-3 **(E)** and CA-HPV-10 **(F)** cells. Over-expression of TGase-4 in PC-3 cells markedly increased the pace of matrix adhesion compared with the control cells **(E)**. In contrast, knocking down TGase-4 marked reduced the adhesiveness.

We previously reported, sublines of CA-HPV-10, which expressed highl levels of TGase-4, were transfected with the anti-TGase-4 ribozyme transgene. Cells which had virtually lost the TGase-4 transcript as the result of the transgene, were selected and verified. These cells have been named CA-HPV-10^ΔTGase4^. PC-3 cells which were largely TGase-4 negative, were transfected with TGase-4 expression vector. Stably transfected cells were established and over-expression of TGase-4 in the cells verified, the cells now termed – PC-3^TGase4exp^ (Figure 1A). It was interesting to observe that expression of TGase-4 had little bearing to the growth rate of both cells (Figure 1B).

### The nature of TGase-4 expression is linked to the adhesion properties of prostate cancer cells

Over-expression of TGase-4 in PC-3 prostate cancer cells increased the adhesiveness to matrix (Figure 1C), accompanied by an increase in matrix invasion of the cells. Of the two over-expressing sublines, PC-3^TGase4exp3^ and PC-3^TGase4exp13^, PC-3^TGase4exp3^ had a more profound effect on matrix adhesion and was used in subsequent experiments. Likewise, knockdown TGase-4 from CA-HPV-10 prostate cancer cells decreased the adhesion and invasion [Figure 1D: * p < 0.05 vs no HGF, ** p < 0.05 vs control cells, by non-paired *t* test]. The same was reflected in the ECIS adhesion assay as shown in Figures 1E and 1F, in that knocking down TGase-4 from CA-HPV-10 dramatically reduced the resistance (31.17 ± 25.1Ω) compared with control [p = 0.042, by non-paired *t* test] (Figure 1F). In contrast, over-expressing TGase-4 in PC-3 resulted in an increase in the electrical resistance (Figure 1E).

### The potential role for integrin and FAK in TGase-4 mediated cell adhesion

TGase-4 associated cell adhesion and cellular motion was highly dependent on integrin. Anti-ß1-integrin was found to reduce the cell matrix adhesion of control PC-3 cells by 27.6% (p = 0.17, by non-paired *t* test) using an ECIS analysis. However, PC-3 cells over-expressing TGase-4 showed a 53.9% dramatic reduction in the adhesion by anti-ß1 integrin antibody (8.92 ± 3.3Ω for PC-3^TGase4exp^ control antibody vs 4.1 ± 1.82Ω for PC-3^TGase4exp^ with anti-ß1 integrin antibody, p = 0.04, by non-paired *t* test).

**Figure 2:**
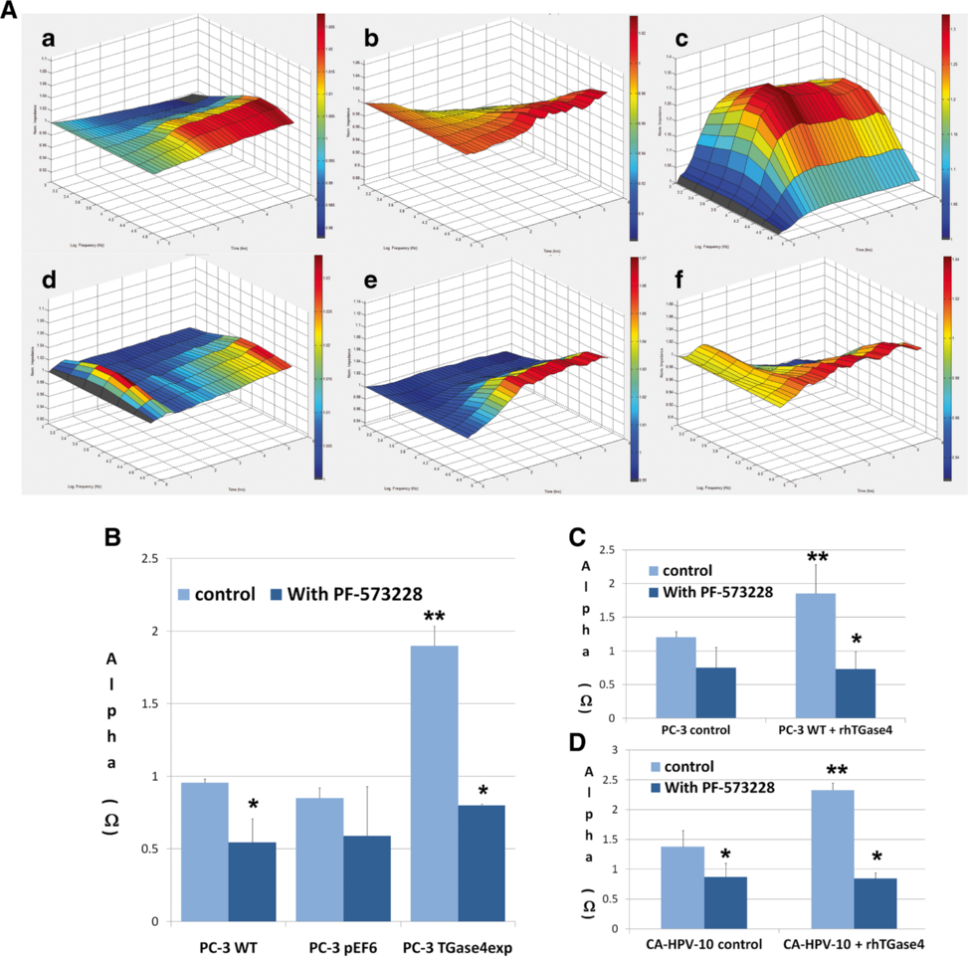
**FAK small inhibitor and cell-matrix adhesion. A**: Effect of FP-573228 on the adhesion of genetically modified PC-3 cells. **a**/**b**/**c**: cell treated with medium alone; **d**/**e**/**f**: cells treated with FP-573228 (5nM). All three cells responded to the FAK inhibitor FP-573228 by reducing the adhesiveness. The inhibitory effect is particularly strong with PC-3^TGase4exp^ cells. **B**: Adhesion as calculated using the Rb model. **C**: The effect of FP-573228 on exogenous TGase-4 induced adhesion in PC-3 control cells. rhTGase-4 significantly increased the adhesiveness which was reverted by FP-573228. **D**: The effect of FP-573228 on exogenous TGase-4 induced adhesion in CA-HPV-10. * p < 0.05 vs no-treatment control of the same cell; ** p < 0.05 vs wile type and control cells.

To further evaluate the signalling events in TGase-4 mediated matrix adhesion, we used a selective small inhibitor to FAK. Here, we evaluated PC-3 cells under the following settings: comparing the response of PC-3 and PC-3^TGase4exp^ to the FAK inhibitor, and comparing the response of PC-3 and CA-HPV-10 control cells to the FAK inhibitor in the presence and absence of exogenous TGase-4 (rhTGase-4). Shown in Figure 2A are ECIS based cell adhesion assay modelled using the Rb method. Compared with the wild type and the control cells, PC-3^TGase4exp^ cells showed a rapid and significant increase in cell adhesion. The data from these experiments are shown in Figure 2B. All three cells responded to the FAK inhibitor FP-573228 by reducing the adhesiveness. The inhibitory effect is particularly strong with PC-3^TGase4exp^ cells. Exogenous rhTGase-4 significantly increased the adhesiveness in both PC-3 and CA-HPV-10 cells (Figure 2C/D). This increase in response to rhTGase-4 was significantly reverted by FP-573228.

### Exogenous TGase-4 induced activation of focal adhesion kinase and paxillin

**Figure 3:**
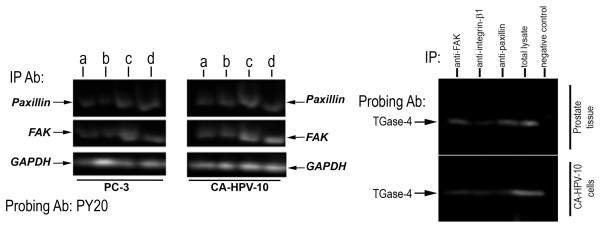
**TGase-4 and the activation of the focal adhesion complex. LEFT**: effect of exogenous TGase-4 on the activation of focal adhesion proteins. Cells were first subject to serum hunger for 2 hours, before treated with: ***a***-serum free medium; ***b***-serum free medium with BSS/BSA buffer; ***c***-rhTGase-4 100 ng/ml and ***d***-sodiium orthovanadate (with 0.1% hudrogen peroxide). After one hour, cells were pelleted and lysed. Anti-FAK or anti-paxillin antibody was added to the protein lysate. Immunoprecipiate was separated on 8% SDS-PAGE gel and probed by PY20 antibody. Total cell lysate used for immunoprecipitation were probed by anti-GAPDH antibody as a load control. rhTGase-4 induced phosphorylation of FAK particular in PC-3 cells, and also induced phosphorylation of paxillin. **RIGHT**: Interaction between TGase-4 and focal adhesion complex proteins. Proteins from fresh frozen human prostate tissues (top) and from a TGase-4 positive CA-HPV-10 cells (bottom) were precipitated with anti-FAK, anti-integrinb1, anti-paxillin or irrelevant antibody (negative control). The immunoprecipitates or total protein lysate from prostate tissues (total lysate, used as a positive control) were separated by SDS-PAGE. TGase-4 was probed with an anti-TGase-4 monoclonal antibody (MaxPaB, Abnova). FAK and paxillin were strongly associated with TGase-4, compared with integrin.

To evaluate the activation status of focal adhesion complex, cells were treated with exogenous rhTGase-4. Figure 3 (left) shows the phosphorylation on tyrosine residues of FAK and paxillin in PC-3 (left) and CA-HPV-10 (right) cells. The low grade of tyrosine phosphorylation of FAK in both cells was markedly activated by rhTGase-4. The same, but to a lesser degree, activation of paxillin was also seen. To further explore the relationship between the focal adhesion complex proteins and TGase-4, immunoprecipitation was carried out. Both FAK and paxillin were found to be able to precipitate TGase-4 from both prostate tissue protein lysate and from CA-HPV-10 cells which were TGase-4 positive (Figure 3 right). It is interesting to observe that interaction between TGase-4 and beta1-integrin was weaker than that with FAK and paxillin (Figure 3 right). Together, it suggests that FAK is a key downstream event in TGase-4 activated cell-matrix adhesion.

### TGase-4 core domain was critical in TGase-4 mediated cell-matrix adhesion

**Figure 4:**
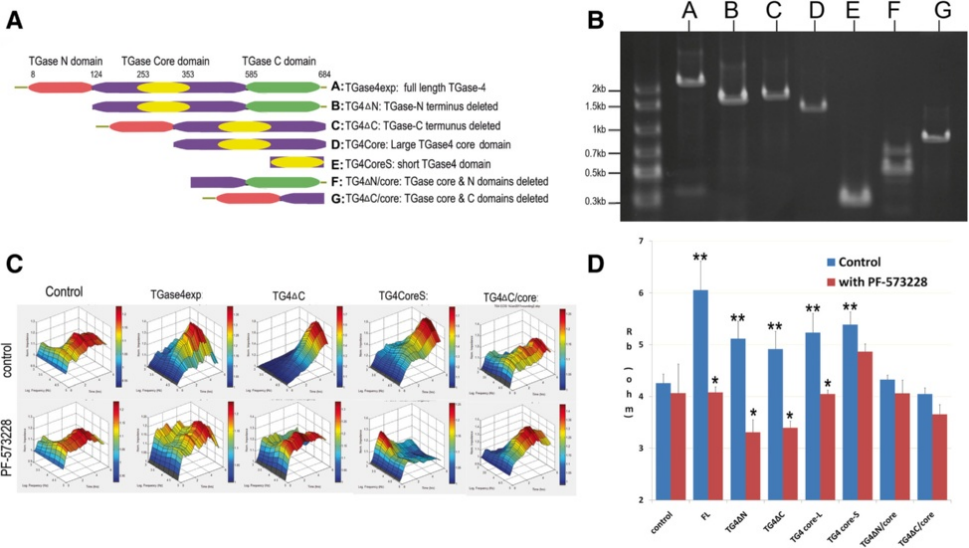
**TGase-4 protein domains and cell-matrix adhesion.** PC-3 cells were transfected with a series of plasmids that coded mutant TGase-4 with either TGase-N domain deleted, N- and core- domains deleted, TGase-C domain deleted, C- and core-domains deleted, the short core domain or the large core domain (**A** and **B**). Cell adhesion after transfection was determined using ECIS in the absence or presence of PF-573228 (**C** and **D**). PC-3 transfected with core domain containing domains (**B**/**C**/**D**/**E**) showed increased matrix adhesion, similar to that seen in cells transfected with the full length TGase-4. Cells transfected with plasmids in which core domains (**F**/**G**) were deleted did not differ from control cells. Increased adhesion mediated by TGase-4 full length, and core domains was inhibited by PF-573228. * p < 0.05 vs no-PF573228 control, ** p < 0.05 vs transfection control.

To further understand the nature of TGase-4 mediated matrix adhesion, PC-3 cells were transfected with plasmids that coded different domains of TGase-4 protein (Figure 4A/B). All 4 sublines that expressed TGase-4 core domains, namely, PC3^TG4ΔN^, PC3^TG4ΔC^, PC3^TG4CoreLarge^ and PC3^TG4CoreSmall^, showed a significant increase in matrix adhesion (Figure 4C/D), similar to that seen with the cell over-expressing full length TGase-4. It is interesting to observe that PC-3 cells expressing only Core domains had a significant effect, whereas cells transfected with N- or C-domains but without the core domain (PC3^TG4ΔC/core^ and PC3^TG4CoreLarge^) did not have this effect. Finally, TGase-4, TGase-4 core-domain mediated matrix adhesion was abolished by PF-573228 (Figure 4C/D).

### TGase-4 expression, localisation and co-localisation of FAK, paxillin and integrin-1 in prostate cancer cells

**Figure 5:**
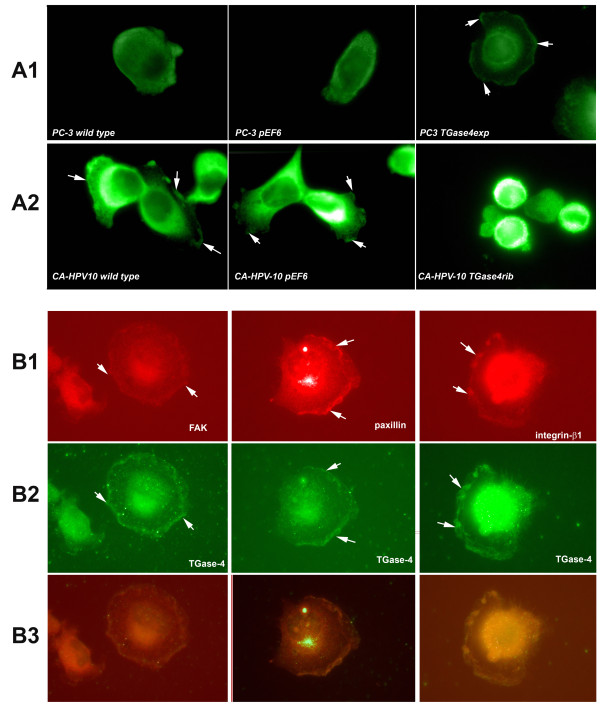
**Co-staining of TGase-4, FAK and paxillin in prostate cancer cells. A**: Immunofluorescence staining of FAK in PC-3 **(A1)** and CA-HPV-10 **(A2)** cells. Wild type and the transfection control PC-3 cells showed little staining of focal adhesion complex. However, TGase-4 transfected (PC3 TGase4exp) spread well over the matrix and displayed a characteristic staining of focal adhesion complex using the anti-FAK antibody. **B**: PC3-TGase4exp cells: Localisation FAK (**B1** left), paxillin (**B1** middle), integrin-β1 (**B1** right) and TGase-4 **(B2)**, and co-localisation of FAK/paxillin/integrin with TGase-4 **(B3)**. All images were from PC-3 cells transfected with TGase-4 (PC-3 TGase4exp). FAK/paxillin/integrin demonstrated a clear co-localisation with TGase-4 in the cells.

In the light of the changes of cell-matrix adhesion after over-expressing TGase-4 in the cells and the change of their response to the FAK inhibitors, we went on to test the pattern of FAK staining in these cells. Shown in Figure 5A1, PC-3 cells, when over expressing TGase-4, exhibit enhanced staining of the focal adhesion complexes. In contrast, CA-HPV-10 wild type and transfection control cells also had a clear pattern of FAK staining. This was diminished after losing TGase-4 (CA-HPV-10/ΔTGase4).

The staining of key components of a focal adhesion complex, FAK, paxillin and integrin was further assessed in PC-3/TGase4exp cells. Shown in Figure 5B are the staining of the individual protein and their merge images. It is clear that FAK, paxillin and beta1-integrin co-localised with TGase-4 in the cells.

### Expression of TGase-4 linked to the in vivo growth of prostate tumours and the colocalisation of FAK, paxillin and integrin-1 in prostate tumour tissues

**Figure 6:**
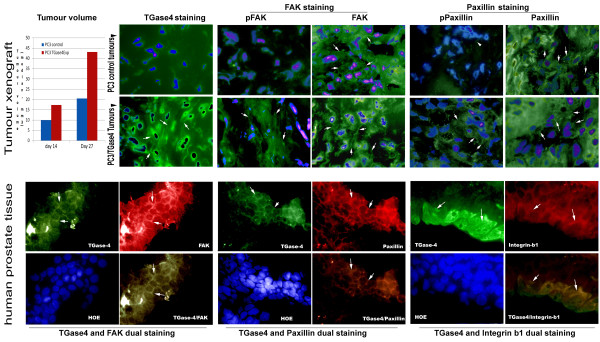
**Effect of TGase-4 on tumour growth *in viv*o and localisation and co-localisation of TGase-4 and the focal adhesion complex proteins. TOP panel**: far left - TGase-4 and *in vivo* growth of tumours. PC-3 over-expressing TGase-4 cells grow significantly faster than control cells (shown are medians). IFC panels: immunofluorescence staining of TGase-4, phosho-FAK, FAK, phospho-Paxillin and Paxillin (left to right) in control and TGase-4 expression PC3 tumour xenograft. TGase-4: Tumours from PC3/TGase4 cells displayed staining of TGase-4 in the cytoplasmic region as well as in the cell peripheries (arrows). Control tumours were negative for TGase-4 staining and mostly missing pFAK and pPaxillin staining, although they are positive for FAK and Paxillin. PC3/TGase4 were seen to stain both pFAK and pPaxillin as well as stain strongly for total FAK and Paxillin (arrows). **BOTTOM panel**: Immunofluorescence co-staining of FAK (left), paxillin (middle) and β1-integrin (right) with TGase-4 in human prostate tissues. Shown in each panel are the match images of FAK/paxillin/integrin, TGase-4 and nucleus staining (Hoescht33528), and the merged image between FAK, paxillin, or integrin and TGase-4. TGase-4 staining appeared in both cells and matrix. There co-localisations of the FAC proteins and TGase-4 were seen in the tissues.

Using athymic nude mice model, it was shown that prostate cancer cells over-expressing TGase-4 had a significantly faster rate of growth [p = 0.05, by two-way ANOVA on rank] (Figure 6, top left).

From the PC-3 tumour xenografts, we stained TGase-4, FAK and Paxillin using phosphospecific antibodies. As shown in Figure 6 [top right panel]. TGase-4 expressing tumours had a positive staining of TGase-4 in the cytosol and at the cell periphery (arrows). Here, we observed an interesting pattern in which both total FAK and total Paxillin were positively stained in the tumour cells in control tumours and in TGase-4 expressing tumours. However, it is very interesting to observe that phospho-FAK and phospho-Paxillin are clearly seen in TGase-4 expressing tumours and virtually invisible in control tumours (Figure 6 top right).

Similarly, we observed co-staining all three FAC proteins, FAK, paxillin and integrin with TGase-4 in a panel of frozen sections of human prostate tissues. Shown in Figure 6 are some representative images. Previously indicated and suspected from the nature of TGase-4, TGase-4 staining can be seen both intracellularly and in the matrix (Figure 6 bottom panel). All three FAC proteins are clearly detected in the tissues, and have a high degree of co-localisation with TGase-4 (Figure 6 bottom panel, composite panels).

## Discussion

In the present study, we examined the possible relationship between TGase-4, a prostate specific transglutaminase-4, and cell-matrix adhesion of prostate cancer cells and have shown an important biological link between TGase-4 and the focal adhesion complex (FAC), namely FAK and paxillin in prostate cancer cells and prostate tissues. We view that these findings have important biological implications in prostate cancer and in the design of therapies of prostate cancer.

The finding that TGase-4 is associated with cell-matrix adhesion is not totally surprising. First, previous reports have shown that TGase-4 is connected with the migration and invasiveness of prostate cancer cells [6,7], two cellular functions closely linked to cell-matrix adhesion; second, other members of the TGase family, namely TGase-2 has also been shown to regulate cell-matrix adhesion. Thus, TGase-4 appears to share the function with TGase-2 in regulating matrix adhesion of prostate cancer cells. However, how TGase regulates the matrix adhesion is not entirely clear. Here, we first showed that blocking FAK by small inhibitor can block TGase-4 mediated cell-matrix adhesion, suggesting that TGase-4 may well affect focal adhesion complex.

The structure of TGase-4 is interesting. The protein has a N- and C-TGase domains as well as a TGase core domain. Transglutaminases has been shown to contain fibronectin binding domain [23]. The later is particularly interesting. Indeed, the present study has found that a neutralising antibody to integrin-β1 can abolish TGase-4 induced cell-matrix adhesion. Integrin-β1 interacts with the extracellular matrix, including fibronectin. Together, it is suggested that TGase-4 may interact with integrin and initiate the cell-matrix adhesion sequences. This is indeed confirmed by the observation that exogenous TGase-4 can induce the phosphorylation of both FAK and paxillin. In deciphering the contribution of different domains to the matrix adhesion, we discovered that the matrix adhesion activities of TGase-4 reside in the TGase-4 core domain, as all the constructs coded core domains have matrix adhesion promoting actions, whereas deleting the core domain from TGase-4 eliminated this activities. The TGase-4 core domain shares a high amino acid homology with the core domains of TGase-1 and TGase-2. TGase-2 has been shown to be a key matrix regulator in cells. Together, TGase-4 core domain plays a central role in TGase-4 mediated cell matrix adhesion in prostate cancer cells. Collectively, the present study shows that TGase-4 may directly activate the cell-matrix adhesion sequence and increase the adhesiveness of prostate cancer cells.

Although TGase-4 has been discovered for over a decade, its pattern of distribution in the prostate gland is not clear, with matrix and cellular distribution being indicated [4,24,25]. The present study and recent literature have shown that TGase-4 is stained in both extracellular matrix and intracellularly. It is also noteworthy that both in cell culture and in prostate tissues, TGase-4, FAK, Paxillin and integrin showed a pattern of co-localisation. This is interesting as it indicates that the close proximity of these proteins may present a mechanism by which over-expression of TGase-4 in prostate cancer tissues may increase the matrix adhesiveness of prostate cancer cells. This is strongly supported by the observation that TGase-4 positive xenografts had activated FAK and Paxillin (pFAK and pPaxillin positive staining) on comparison to control tumours in which FAK and Paxillin were present but remain less active (less pFAK and pPaxillin staining). This finding is highly interesting and has not been reported with other transglutaminases, although it has been indicated that FAK may be involved in the induction of tissue transglutaminses by hyaluronic acid [26] Presently, although the String search has predicted a possible interaction between TGase-4 and vimentin, the function[s] of the intracellular TGase-4 is not known and warrants further investigation (Additional file 1).

The connection between TGase-4 and cell-matrix adhesion is very interesting from a therapeutic point of view. Already shown in the present study, inhibitor to FAK is able to revert TGase-4-induced matrix adhesion of prostate cancer cells. Genetic manipulation of FAK can inhibit tumour growth [27]. FAK inhibitor is presently in clinical trials in treating a number of human solid tumours [28-30]. Although the inhibitor is yet to be trialled in human prostate cancer, the present study clearly shows that FAK inhibitor may have an important implication in the treatment of prostate cancer and that the levels of TGase-4 in prostate cancer may be one of the determining factors to the sensitivity of the patients to FAK inhibitor.

In conclusion, Prostate Transglutaminase, TGase-4, a protein uniquely expressed in human prostate gland, plays an important role in mediating cell-matrix adhesion of prostate cancer cells. This effect is possibly mediated by the Core domain of the protein and requires the participation of integrin medicated focal adhesion kinase pathway. The findings have an important implication in devising treatment in prostate cancer. For example, the levels of TGase-4 may be a factor in deciding the response of a patient to FAK inhibitors as well as itself being a therapeutic target. The full clinical implication of TGase-4 is now open for investigation.

## Competing interests

The authors declare that they have no competing interests.

## Authors’ contributions

WGJ contributed to study design, experimental work and manuscript preparation. YL contributed to experimental design, experimental work and manuscript preparation. FR and AJS contributed to in vivo and immunohistochemical work. RJA contributed to study design and manuscript preparation. MDM contributed to study design and manuscript preparation. HGK contributed the collection of human prostate tissues. All authors read and approved the final manuscript.

## Supplementary Material

Additional file 1 PCR primers used in the study. Click here for file
