# Supplementary material for: Prostate transglutaminase (TGase-4, TGaseP) enhances the adhesion of prostate cancer cells to extracellular matrix, the potential role of TGase-core domain
Source: J Transl Med. 2013 Oct 25;11:269. doi: 10.1186/1479-5876-11-269 (PMC3874635; doi:10.1186/1479-5876-11-269)
Supplement: Additional file 1 — PCR primers used in the study. [file 1479-5876-11-269-S1.pdf]

Supplement-1. primers used in the study

	**Sense(5' –'3)**	**AntisSense (5' – '3)**
TGase-4 expression	Atgatggatgcatcaaaaga (TGase4ExFa3)	Ctacttggtgatgagaacaatcttctga, TGase4ExRa2
TGase-4 (position 62)	atggatgcatcaaaagagc	Aggtgaaacacctgtcctc Aactgaacctgaccgtacaaggtgaaacacctgtcctc, for Q-PCR))
TGase-4(position 1957)	ataaaatgcaccccaataaa	Ctacttggtgatgagaacaatc (actgaacctgaccgtacacctacttggtgatgagaacaatc, for Q-PCR)
TGase-N deletion	atgcacatccttaagtctgaagaaaa	cttggtgatgagaacaatctt
TGase-C deletion	Atgatggatgcatcaaaagag	gtactggaaagacgtgaatactt
TGase-core only	Atgaaccacatccttaagtctgaagaa	Gtactggaaagacgtgaatact
TGase-central core	ATGCaggctgtgtgctttgg,	Cggcgttgcgtccac
TGase-4 DelN/core	Atgatggatgcatcaaaagagctgca	cgctcctgcggcagcctgctt
TGase-4DelC/core	gaagcaggctgccgcagga	Ctacttggtgatgagaacaa
Gase-4 ribozyme1	Atgatggatgcatcaaaagag	Ctacttggtgatgagaacaa
TGase-4 ribozyme1	ctgcagtttggaactcccatgtgtggtgactgatgagtccgtgagga	actagttgaatcaggacaacgccgtttttcgtcctcacgga
TGase-4 ribozyme2	ctgcagttcagttggtggtggtagctgatgagtccgtgagga,	actagtccagcccctacaatttcgtcctcacggga,
GAPDH	agcttgtcatcaatggaaat	cttcaccaccttcttgatgt
GAPDH for Q-PCR	ctgagtacgtcgtggagtc	Actgaacctgaccgtacacagagatgatgaccctttttg
